# Class-aware multi-source domain adaptation algorithm for medical image analysis using reweighted matrix matching strategy

**DOI:** 10.1371/journal.pone.0323676

**Published:** 2025-07-23

**Authors:** Huiying Zhang, Yongmeng Li, Lei He, Wenbo Zhang, Yuchen Shen, Lumin Xing

**Affiliations:** 1 Department of Thoracic Surgery, Qilu Hospital of Shandong University, Jinan, Shandong, China; 2 The First Affiliated Hospital of Shandong First Medical University & Shandong Provincial Qianfoshan Hospital, Jinan, Shandong, China; 3 Shandong First Medical University & Shandong Academy of Medical Sciences, Jinan, Shandong, China; University of Mosul, IRAQ

## Abstract

Multi-source domain adaptation leverages complementary knowledge from multiple source domains to enhance transfer effectiveness, making it more suitable for complex medical scenarios compared to single-source domain adaptation. However, most existing studies operate under the assumption that the source and target domains share identical class distributions, leaving the challenge of addressing class shift in multi-source domain adaptation largely unexplored. To address this gap, this study proposes a Class-Aware Multi-Source Domain Adaptation algorithm based on a Reweighted Matrix Matching strategy (CAMSDA-RMM). This algorithm employs a class-aware strategy to strengthen positive transfer effects between similar classes. Additionally, first-order and second-order moment matching strategies are applied to effectively align the source and target domains, while an adaptive weighting mechanism is utilized to optimize the contributions of different source domains to the target domain. These approaches collectively improve classification accuracy and domain adaptability. Experimental results on four publicly available chest X-ray datasets demonstrate that the superiority of the proposed method.

## 1. Introduction

Transfer learning has emerged as a critical technique for addressing knowledge transfer challenges across different domains in medical imaging and has garnered considerable attention in recent years [1]. In multi-source domain settings, the issue of class shift becomes particularly prominent due to significant class inconsistencies among different source domains [2]. Class shift refers to the mismatch in class distributions between the source and target domains, which limits the accuracy of traditional single-source domain adaptation methods. To overcome these limitations, extensive research has been conducted globally to develop multi-source domain adaptation techniques. These efforts aim to more effectively address class shift issues and ensure the efficient transfer of knowledge across domains [3].

Traditional domain adaptation achieves knowledge transfer by minimizing the difference between different data distributions, and then uses existing knowledge to identify unknown knowledge [4,5]. Early studies in this area, mostly focused on single-source adaptation, can be categorized into three main approaches [6]: (1) Discrepancy-based methods [7]: Long et al. introduced a joint adaptation network for deep transfer learning, which aligns the distributions of data and classifiers across domains to address misclassification issues commonly encountered in transfer learning [8]. Wang et al. embedded the source and target image information into a manifold and then classified the target image class by aligning the distributions of the two [9]. Zhang et al. embedded the distributions of the source and target domains in the kernel space and effectively aligned the two different distributions [10]. (2) Adversarial learning methods: Hoffman et al. [16] introduced an adversarial mechanism to reduce the overall distance between distributions of different domains, but ignored the influence of class factors [11]. (3) Consistency-based methods: Kumar et al. learned more stable and discriminative features by placing the decision boundary in low-density regions, aligning distributions in each feature space independently [12]. While these approaches have achieved progress in reducing domain shift, they are generally designed for single-source settings and struggle to settle the complexities of multi-source domains [13].

In the context of multi-source domain adaptation, this approach effectively integrates multiple source domains, addressing the existing limitations, where sample diversity is often insufficient. However, multi-source domain adaptation introduces new challenges, primarily involving significant domain shifts and varying contributions [14]. To address domain shifts, Zhao et al. employed adversarial learning to capture invariant and discriminative feature representations across multiple domains [15], while Peng et al. designed a method based on moment matching. Nevertheless, these methods tend to treat all source domains equally, overlooking their varying contributions to the target task [16]. To account for this, Xu et al. minimized domain shifts in adversarial learning by calculating domain relevance [17], while Zhu et al. introduced a multi-feature space transfer framework aimed at aligning domain-specific distributions and classifiers, significantly minimizing misclassification [18]. Zhang et al. introduced a multi-source selection method that selects source domains most similar to the target domain through strategies such as nearest-neighbor and weighted selection [19]. Although these approaches have made progress in reducing domain shifts and addressing the unequal contributions of source domains, most assume complete class overlap among domains. However, class shift remains an underexplored issue in multi-source domain adaptation, where misalignment can negatively affect overall adaptation performance. Therefore, further research is urgent for multi-source domain adaptation methods that effectively address class shifts and improve performance. To tackle class shift, Zhu et al. introduced an attention-based domain adaptation algorithm that prioritizes source domains more closely aligned with the target domain by assigning them higher weights, ensuring that the feature extractor generates aligned and discriminative visual representations [20]. However, this approach only mitigates the negative transfer caused by class shift at the domain level. Wang et al. leveraged optimal transport theory to handle multi-source domain heterogeneity and achieve fine-grained sample-level alignment between sources and the target domain [21]. Nonetheless, optimal transport computation is typically resource-intensive, especially when dealing with high-dimensional data across multiple domains. Sahoo et al. developed a peer-assisted learning classifier for partial domain adaptation, aiming to minimize negative transfer by synchronizing the distributions between the overlapping source domains and the target domain [22].

Building on prior works, our work designs a class-aware multi-source domain adaptation algorithm based on reweighted moment matching (CAMSDA-RMM). This approach focuses on identifying source classes pertinent to the target, boosting their transfer while minimizing the influence of unrelated classes. Additionally, first- and second-order moment matching strategies are employed to effectively align different domains, with an adaptive weighting mechanism optimizing the contribution of each source domain. This holistic strategy facilitates a more accurate management of class shifts in multi-source domain adaptation, enhancing adaptability and classification performance in the target domain. The majority work can be concluded as:

1. A class-aware strategy is proposed to selectively enhance the transfer of classes relevant to the target domain, effectively addressing class shift.
2. A reweighted moment matching method is introduced to align the first- and second-order statistical moments among different domains, significantly reducing distribution discrepancies.
3. An adaptive weighting mechanism is developed to dynamically adjust the influence of each source domain based on its relevance, optimizing overall adaptability.

## 2. Problem definition and theoretical analysis

### 2.1 Problem definition

**Fig 1:**
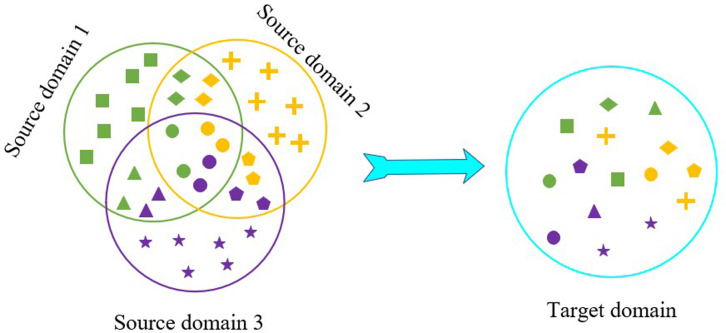
Illustration of the class shift problem in multi-source domain adaptation.

One of the key obstacles in multi-source domain adaptation is the phenomenon of class shift. In this scenario, the target domain only overlaps with a limited number of classes from each source domain, while each source domain has its own exclusive classes. This scenario is more reflective of real-world applications. Fig 1 illustrates the sample distributions: the left side displays the distributions from three source domains, while the right side depicts the distribution of the target. Different shapes represent different sample classes: the unique class of Source Domain 1 is represented by “square,” Source Domain 2 by “cross,” and Source Domain 3 by “star.” Shared classes such as “rhombus,” “triangle,” “pentagon,” and “circle” represent the common classes across source domains. The color variations indicate samples from different source domains.

In this scenario, with *N* source domains $\left\{{𝒟}_{S_{1}},{𝒟}_{S_{2}},\cdots ,{𝒟}_{S_{N}}\right\}$ and a target domain ${𝒟}_{T}$, the data distributions of the source and target domains are represented as ${\left\{{𝒫}_{S_{i}}\right\}}_{i=1}^{N}$ and ${𝒫}_{T}$, respectively. The goal of multi-source domain training is to leverage all the source domain data ${\left\{(X_{si},Y_{si})\right\}}_{i=1}^{N}$ along with the unseen target domain data $X_{t}={\left\{x_{t}^{j}\right\}}_{j=1}^{N_{t}}$ to train a model capable of accurately predicting the target domain labels $X_{t}={\left\{x_{t}^{j}\right\}}_{j=1}^{N_{t}}$. Although the labels in different source domains may vary, the union of all source domain labels forms the target domain label space.

### 2.2 Theoretical analysis

To effectively address the class shift issue in multi-source domain adaptation, this paper proposes CAMSDA-RMM. A detailed theoretical analysis is provided from two perspectives: the class-aware strategy and the reweighted moment matching strategy.

The core idea of the class-aware strategy is to identify and selectively enhance the classes relevant to the target domain while minimizing interference from irrelevant classes. The theoretical foundation for this approach is based on several key points: At the heart of the class shift issue is the mismatch between the class distributions of the source domains and those of the target domain. A strategy that can differentiate between relevant and irrelevant classes is needed to prevent interference from irrelevant classes during the transfer process. Second, the classes related to the target domain are of greater value during transfer, as their features contribute more significantly to the target task. By identifying these classes and increasing their weights, the important features can be fully utilized in the transfer process, thereby improving classification accuracy and adaptability. Finally, irrelevant classes can introduce noise during the transfer process, reducing the overall performance of the model. By identifying and minimizing the impact of these irrelevant classes, the noise can be reduced, improving the model’s robustness and ability to generalize.

The core idea of the reweighted moment matching strategy is to achieve more precise alignment through reweighting the first- and second-order statistical moments. The theoretical foundation of this approach includes the following points: First, to achieve effective transfer, the distribution discrepancy of different domains must be minimized. Traditional distribution alignment methods often focus solely on aligning first-order statistical moments, neglecting higher-order moments, which may result in suboptimal alignment. Second, higher-order statistical moments contain additional structural information about the data distribution, which is crucial for achieving more precise distribution alignment. Therefore, aligning both first- and second-order moments can more comprehensively reduce the distribution discrepancies. Finally, due to differences in similarity among the various source domains, the contribution of knowledge transfer varies. Therefore, reweighted moment matching strategy is introduced to quantify the contributions of different source domains and assign corresponding weights, achieving more effective transfer results.

## 3. Methodology

### 3.1 Class-aware strategy

A major hurdle is the effective alignment of the target domain with various source domains, particularly when there are pronounced shifts between the domains. Real-time perception of shared categories between source and target domains is a key behavior to promote knowledge transfer. To address this, a class-aware strategy is proposed. This strategy focuses on identifying and reinforcing the connection between similar classes in the target and source domains to promote positive transfer while suppressing negative transfer from dissimilar classes, thus effectively reducing the adverse effects caused by class shift.

Class shift arises from differences in the classes present in each source domain, which can impact the model’s transferability. Specifically, given multiple source domains, each containing a distinct set of classes, let the source class sets are defined as $Q_{s}$, and the target class sets are defined as $Q_{t}$. Class shift can thus be defined as the divergence between the source and target class sets. To mitigate class shift, the proposed class-aware strategy is optimized through the following steps:

1) Identifying and reinforcing the shared classes to leverage these for enhancing model transferability.
2) Reducing reliance on classes in source domains that do not exist in target domain, thereby minimizing interference from irrelevant information and alleviating the impact of negative transfer.

Given that the data distributions of each source domain may differ, classes with the same label are treated as distinct. Based on this assumption, source domain labels are redefined. Let *Q* denotes the number of classes, and source domain data $X_{si}(i\in [1,N])$ are relabeled as ${\hat{Y}}_{si}$. The loss function for class-aware training *A* during training is defined as follows:


$${ℒ}_{class}=-E\left[{\hat{Y}}_{S}^{T}\ln Q(X_{S})\right]$$ (2)

The training of class-aware *A* is based on the new label ${\hat{Y}}_{si}$, so that it can accurately identify the source and class of the data. After the training is completed, *A* is used to predict the probability of belonging to each class, thereby obtaining the class relevance vector $w^{c}=\left\{w_{1}^{c},w_{\text{2}}^{c},\cdots ,w_{N}^{c}\right\}$.

### 3.2 Reweighted moment matching strategy

Building on the class-aware strategy, it is crucial to further reduce the impact of class shift on domain alignment. A representative approach is the multi-source domain adaptation moment matching (M3SDA) method [23], which distills features by minimizing the moment distance. However, this method does not account for the varying contributions of source domains, failing to effectively distinguish the specific contribution of each source domain. To address this, a reweighted moment matching strategy is proposed, which calculates the contributions of source domains, thereby optimizing the accuracy and effectiveness of domain alignment.

Specifically, by utilizing the class probabilities predicted by class-aware model *A*, the weighted ensemble predictions for target samples are obtained, leading to varying contribution values. Reweighted moment matching achieves better results by matching the distribution among different source domains and the distribution between the source and the target. For different source domains, the loss function is calculated as follows:


$${ℒ}_{ss-awmd}=\frac{2}{N(N-1)}\sum_{n_{1}=1}^{N-1}\sum_{n_{2}=n_{1}+1}^{N}AWMD\left({𝒟}_{{sn}_{1}},{𝒟}_{{sn}_{2}}\right)=\frac{2}{N(N-1)}\sum_{n_{1}=1}^{N-1}\sum_{n_{2}=n_{1}+1}^{N}\left(w_{i}(\|𝔼\left(ℳ{\left(X_{{sn}_{1}}\right)}^{1}\right)\;-𝔼\left({\;ℳ\left({\left(X_{{sn}_{2}}\right)}^{1}\right)\|}_{2}+\|𝔼\left(ℳ{\left(X_{{sn}_{1}}\right)}^{2}\right)\;-E{\;\left(ℳ{\left(X_{{sn}_{2}}\right)}^{2}\right)\|}_{2}\right)\right)$$ (3)

where 𝔼 represents the mean operation across domains, $ℳ{\left(X_{sn_{1}}\right)}^{1}$ and $G{\left(X_{si}\right)}^{2}$ stand for the first and second moments of features from source domain $s_{i}$, ${𝒟}_{sn_{1}}$ and ${𝒟}_{sn_{2}}$ represent the processed samples on the corresponding source domain, $w_{i}$ represents the contribution weight *w* of the *i*_th_ component, and ${\left\|\cdot \right\|}_{2}$ represents the 2-norm of the feature space.

Moreover, for source and target domains, the following loss function is constructed:


$${ℒ}_{st-awmd}=\frac{2}{N\cdot N}\sum_{n_{1}=1}^{N-1}\sum_{n_{2}=1}^{N}AWMD\left({𝒟}_{{sn}_{1}},{𝒟}_{{tn}_{2}}\right)=\frac{2}{N\cdot N}\sum_{n_{1}=1}^{N}\sum_{n_{2}=1}^{N}\left(w_{i}(\|𝔼\left(ℳ{\left(X_{{sn}_{1}}\right)}^{1}\right)\;-E\left({\;ℳ\left({\left(X_{{tn}_{2}}\right)}^{1}\right)\|}_{2}+\|𝔼\left(ℳ{\left(X_{{sn}_{1}}\right)}^{2}\right)\;-E{\;\left(ℳ{\left(X_{{tn}_{2}}\right)}^{2}\right)\|}_{2}\right)\right)$$ (4)

where $ℳ{\left(X_{ti}\right)}^{1}$ and $ℳ{\left(X_{ti}\right)}^{2}$ represent the first and second moments of the target domain features, ${𝒟}_{sn_{1}}$ and ${𝒟}_{tn_{2}}$ represent the processed samples from the source and target domains, respectively. By integrating the above alignment functions, the overall domain-level alignment loss function is formulated as:


$${ℒ}_{awmd}={ℒ}_{ss-awmd}+{ℒ}_{st-awmd}$$ (5)

The first term constrains the alignment between any two source domains, while the second term reduces the domain shift. Compared to traditional moment matching, the proposed reweighted moment matching offers the following advantages: first, it aligns feature distributions separately; second, it prioritizes alignment with high-contribution source domains to reduce the negative impact of dissimilar domains.

Finally, by measuring the marginal distribution differences between source and target domains, the contribution of each source domain is determined. The *N* contributions are normalized, yielding the adaptive contribution weight $w_{i}$ of the *n*_th_ source domain to the target domain as follows:


$$w_{i}=\frac{w_{a}^{i}}{\sum_{i^{\prime }=1}^{N}w_{a}^{i^{\prime }}}$$ (6)

$$w_{a}^{i}=\frac{1}{AWMD(D_{s_{n}},D_{t})}$$ (7)

where $w_{a}^{i}$ represents the normalization of *N* contributions, and $AWMD(D_{s_{n}},D_{t})$ represents the reweighted moment distance between the $n_{th}$ source domain and the target domain in the feature space.

### 3.3 Proposed framework

The class-aware strategy and reweighted moment matching strategy form the core methodologies for effective alignment. The class-aware strategy enhances the connection between common classes in both domains, optimizing forward transfer and mitigating the negative effects. Additionally, the reweighted moment matching strategy enhances domain alignment by assessing the contribution of each source domain and aligning feature distributions both among the source domains and between the source and target domains. The combination not only optimizes the data transfer learning process but also provides a more precise and efficient framework for multi-source domain adaptation.

**Fig 2:**
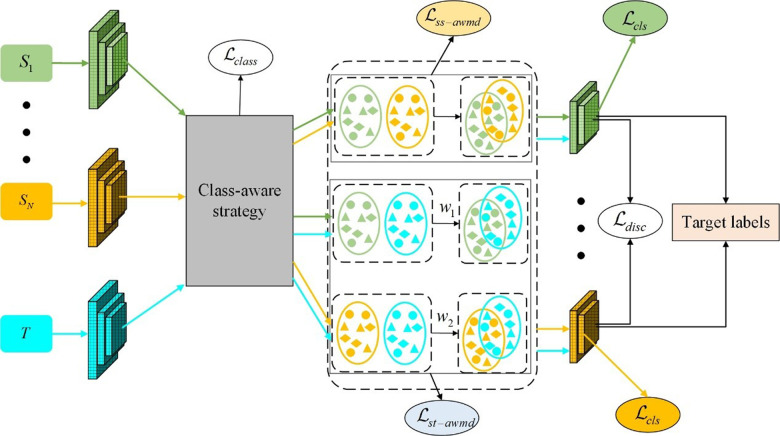
The structure of proposed method.

The components of the proposed algorithm include feature extraction, class awareness, re-weighted matrix matching, and classification, which is depicted in Fig 2, where domains $S_{1}$ to $S_{N}$ represent source domains 1 through *N*, and *T* represents the target domain. ℳ denotes the shared feature extractor. *A* represents the class-aware mechanism, ${ℒ}_{class}$ represents the loss function for class-awareness, *W* denotes the re-weighted matrix matching, $w_{i}$ signifies the adaptive contribution weights in the re-weighting mechanism, ${ℒ}_{ss-awmd}$ and ${ℒ}_{st-awmd}$ stand for the loss functions of feature alignment by only source, and source and target. *C* also represents the domain classifier, and ${ℒ}_{cls}$ represents the domain classification cross-entropy loss.

The complete optimization process of CAMSDA-RMM is detailed in Algorithm 1. The time complexity primarily depends on several key steps: data sampling, feature extraction, loss calculation, and parameter updates. The algorithm runs iteratively for a maximum of *K* iterations, processing *m* images per iteration. The complexity of feature extraction depends on the model specifics, denoted by O(f), while loss calculation and parameter updates have a complexity of O(c). Therefore, the complexity per iteration is O(m*(f+c)), and the total time complexity is proportional to O(K*m*(f+c)). This shows that the runtime is proportional to the number of iterations, the number of images processed per iteration, and the complexity of feature extraction and loss computation.

Algorithm 1: CAMSDA-RMM

Input: Labeled source domain samples, unlabeled target domain samples, maximum number of iterations *K*;

Output: Predicted target domain labels.

1. Randomly sample *m* images ${\left\{(X_{i}^{sj},Y_{i}^{sj})\right\}}_{i=1}^{m}$ from the source domain ${\left\{(X_{si},Y_{si})\right\}}_{i=1}^{N}$;

2. Sample *m* images ${\left\{x_{i}^{t}\right\}}_{i=1}^{m}$ from the target domain $X_{t}$;

3. Feed both source and target samples into the feature extractor *G* to obtain common latent representations $G(x_{i}^{sj})$ and $G(x_{i}^{t})$;

4. Feed the common latent representations of the source samples into the class-aware mechanism to obtain domain-specific representations of the source samples;

5. Perform domain feature identification according to Equation (1) and compute the loss ${ℒ}_{class}$;

6. Construct and compute the adaptive contribution weights $w_{i}$ of the source domains to the target domain based on Equations (2), (6), and (7);

7. Align the features between different source domains according to Equation (3) and compute the loss ${ℒ}_{ss-awmd}$;

8. Align the features between the source and target domains according to Equation (4) and compute the loss ${ℒ}_{st-awmd}$;

9. Pass the domain-specific features of the source samples through the domain-specific classifier to obtain *A*, $C_{j}(G(X_{i}^{sj}))$ and compute the loss according to Equation (8);

10. Compute and update the total objective loss ${ℒ}_{total}$ according to Equation (9);

11. Update the corresponding model parameters by minimizing the loss in Equation (9);

12. Repeat steps 1–11 until the maximum number of iterations *K* is reached or the convergence criterion ${ℒ}_{total}$ is met.

### 3.4 Objective Function

To address the class shift issue in domain adaptation, CAMSDA-RMM considers a composite objective function composed of the class-aware loss function, the domain-level comprehensive alignment loss function, and the cross-entropy loss function. The domain classifier cross-entropy loss is constructed as follows:


$${ℒ}_{cls}=\sum_{j=1}^{N}{𝔼}_{x\sim X_{sj}}J(C_{j}\left(ℳ\left(X_{i}^{sj}\right)\right),y_{i}^{sj})$$ (8)

where *C* represents the output of the integrated *N* domain predictors and J(·) represents the cross-entropy classification loss. The total loss of CAMSDA-RMM is formulated as:


$${ℒ}_{total}={ℒ}_{class}+{ℒ}_{awmd}+{ℒ}_{cls}$$ (9)

where ${ℒ}_{total}$ represents the total objective loss, ${ℒ}_{class}$ represents the class-aware loss, ${ℒ}_{awmd}$ represents the domain-level comprehensive alignment loss, and ${ℒ}_{cls}$ represents the cross-entropy loss for domain classification. Minimizing this total objective loss, composed of the above loss terms, achieves multi-source domain adaptation.

## 4. Experiments and results analysis

### 4.1 Dataset description

This study constructs multi-source domain adaptation scenarios for medical imaging using four publicly available chest X-ray datasets: NIH-CXR14 [24], MIMIC-CXR [25], CheXpert [26], and Open-i [27].

NIH-CXR14 [24] is a large-scale public dataset containing 112,120 chest X-ray images from 30,805 patients. It has been widely used to evaluate computer-aided diagnosis (CAD) algorithms, such as lung nodule detection and pulmonary disease classification.

MIMIC-CXR [25] includes 377,110 images and associated text reports from 227,835 radiological studies conducted at the Beth Israel Deaconess Medical Center in Boston, Massachusetts.

CheXpert [26], released in 2019 by Stanford University researchers, comprises 224,316 X-ray images from 65,240 patients who underwent at least one radiological examination. This dataset is designed to aid researchers in developing automated algorithms for assisting radiologists in disease detection and diagnosis.

Open-i [27], compiled by Indiana University Hospital, collects open-source literature and biomedical images from the web. It contains 3,955 radiology reports corresponding to 7,470 chest X-rays, including frontal and lateral views. To maintain consistency with other datasets, only the 3,955 frontal images are retained for the experiments in this study.

To adhere to the conventional unsupervised domain adaptation setup, a closed set of diseases is selected as multi-class labels across the four datasets: Atelectasis, Cardiomegaly, Effusion, Consolidation, Edema, and Pneumonia. Two multi-source domain adaptation transfer scenarios are constructed for this study: (1) NIH-CXR14, CheXpert, and MIMIC-CXR to Open-i. (2) NIH-CXR14 and CheXpert to Open-i.

### 4.2 Experimental settings

To address potential differences in image sizes and aspect ratios across datasets, all images are resized to 128 × 128 before being fed into the network. This resizing ensures a consistent input size across datasets and reduces variability between them. To expand the training set, two simple yet effective data augmentation techniques are employed: random cropping and horizontal flipping. Random cropping allows the network to learn features at different scales by applying random cropping operations to the images. Horizontal flipping introduces additional diversity to the training data by horizontally flipping the images.

All experiments are conducted using the PyTorch framework on an NVIDIA 3090 GPU. The optimization process utilized stochastic gradient descent (SGD) with a weight decay of 0.0005 and a momentum of 0.9. The initial learning rate is set to 0.001. Three sets of experiments are designed for this study:

 Experiment 1: Comparison of CAMSDA-RMM with existing domain adaptation methods: This set includes comparative analysis with both single-source and multi-source domain adaptation methods to evaluate the effectiveness of the proposed algorithm.

 Experiment 2: Effectiveness of the class-aware strategy: This experiment verifies the capability of the class-aware strategy to identify and enhance the relationships between similar classes in the source and target domains.

 Experiment 3: Effectiveness of the reweighted matrix matching strategy: This analysis demonstrates how the reweighted matrix matching strategy quantifies the varying contributions of different source domains to the classification task in the target domain, optimizing the accuracy and effectiveness of domain alignment.

### 4.3 Experimental comparison

CAMSDA-RMM is compared with existing domain adaptation algorithms The compared methods include the single-source algorithm “Single Best,” which represents the best performance, and multi-source algorithms such as JAN [8], DCTN [18], ABMSDA [20], CASR [21], TSCDA [22] and M3SDA [23]. ResNet-50 is employed as the pre-trained feature extractor, and a *Softmax* classifier is used to initialize the model. The results are presented in Table 1 and Table 2.

**Table 1:** Comparative analysis of CAMSDA-RMM with existing domain adaptation methods in the first transfer scenario.

Type	Method	AT	CA	EF	CO	ED	PN	Avg
Single source	Single Best	63.37	90.50	88.71	63.54	82.44	77.30	77.64
Multi-source	JAN	65.88	97.21	95.42	75.27	86.55	75.11	82.57
	DCTN	70.53	96.23	92.81	77.61	86.77	86.88	85.14
	M3SDA	69.76	98.58	95.23	78.56	87.56	83.60	85.55
	ABMSDA	70.76	**98.98**	94.23	75.56	87.76	89.25	86.09
	CASR	89.99	98.70	**98.30**	85.95	**95.80**	82.86	91.93
	TSCDA	89.38	98.32	96.25	86.72	94.30	90.37	92.56
	CAMSDA-RMM	**90.50**	98.02	97.82	**88.80**	95.40	**90.44**	**93.50**

**Table 2:** Paired *t*-test results for the first transfer scenario.

Method	*t-*statistic	*p-*value
Single Best	5.521	0.0015
JAN	3.571	0.0118
DCTN	3.650	0.0107
M3SDA	3.128	0.0204
ABMSDA	2.729	0.0342
CASR	1.405	0.2095
TSCDA	3.027	0.0232

**Fig 3:**
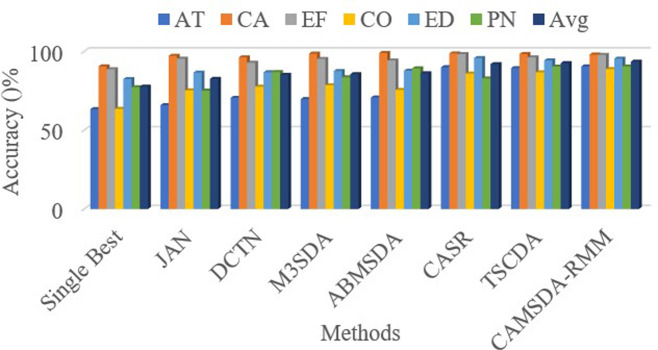
Experimental comparison results in the first transfer scenario.

Table 1 and Fig 3 presents the experimental results for the transfer scenario from NIH-CXR14, CheXpert, and MIMIC-CXR to Open-i. The best results are highlighted in bold for clarity. For convenience, the following abbreviations are used throughout the table: AT for Atelectasis, CA for Cardiomegaly, EF for Effusion, CO for Consolidation, ED for Edema, and PN for Pneumonia.

The experimental results demonstrate that CAMSDA-RMM outperforms other existing domain adaptation methods in terms of average accuracy. Specifically, compared to the Single Best method, which achieved an accuracy of 77.64%, CAMSDA-RMM shows an improvement of 15.86%. This result indicates that multi-source domain adaptation alleviates the issues of limited sample diversity inherent in single-source domain adaptation, thereby significantly enhancing classification performance. Furthermore, CAMSDA-RMM surpasses other existing multi-source domain adaptation methods. As shown in Table 1, CAMSDA-RMM outperforms the multi-source domain adaptation methods JAN, DCTN, M3SDA, ABMSDA, CASR, and TSCDA by 10.93%, 8.36%, 7.95%, 7.41%, 1.57%, and 0.94%, respectively.

Further statistical analysis was conducted on the results presented in Table 1. CAMSDA-RMM exhibited the highest average performance (93.50%), followed by TSCDA (92.56%) and CASR (91.93%). Notably, CAMSDA-RMM demonstrated lower performance variability, as indicated by its relatively low standard deviation (4.08), whereas Single Best exhibited the highest standard deviation (11.95), suggesting significant performance fluctuations. To assess the statistical significance of performance differences between CAMSDA-RMM and other methods, a paired ttt-test was conducted. The results are summarized in Table 2.

A *p*-value below 0.05 indicates a statistically significant difference compared to CAMSDA-RMM. The results show that Single Best, JAN, DCTN, M3SDA, ABMSDA, and TSCDA exhibit significant differences relative to CAMSDA-RMM (*p* < 0.05), whereas CASR does not (*p*  = 0.2095). These findings suggest that CAMSDA-RMM significantly outperforms most baseline methods, particularly Single Best, JAN, and DCTN, while the difference in performance compared to CASR is not statistically significant.

Through experimental validation, the proposed method demonstrates adaptability in the transfer scenario of multi-label medical chest X-ray datasets and can effectively account for the differences between multiple medical image datasets. Additionally, variations in performance across different label categories are observed. In certain categories, the proposed method outperforms others, such as in Atelectasis, Consolidation, and Pneumonia, where it delivers superior results. However, in other categories, such as Effusion and Edema, the performance of CASR surpasses that of the proposed method, and for the Cardiomegaly class, ABMSDA performs better. These findings suggest that distribution differences and label noise across categories significantly affect model performance, highlighting the need for further investigation and exploration. Nevertheless, overall, the proposed method delivers optimal average performance. These results suggest that the proposed method could serve as an effective tool for future multi-label medical image analysis. Table 2 presents the experimental results for the transfer scenario from NIH-CXR14 and CheXpert to Open-i.

The experimental results show that CAMSDA-RMM improves performance by 4.81% compared to the Single Best method, which achieved 86.93%. CAMSDA-RMM outperforms other existing multi-source domain adaptation methods, such as JAN and DCTN, by 1.44% and 1.65%, respectively. Both JAN and DCTN treat all source domains equally during source domain feature extraction, assigning the same weight to each source domain and neglecting the differences in their contributions to the target task. In contrast, the proposed method employs a class-aware strategy, coupled with a reweighted moment matching approach, which quantifies the specific contributions of each source domain to the target domain’s classification task, thereby optimizing domain alignment accuracy and effectiveness. Additionally, CAMSDA-RMM outperforms other multi-source domain adaptation methods such as M3SDA, CASR, and TSCDA by 2.45%, 2.31%, and 0.49%, respectively. This further demonstrates that the proposed reweighted moment matching strategy allows for more precise weighting of each source domain, rather than using a coarse domain-level weight assignment. Consequently, this enhances domain alignment more effectively.

To evaluate the statistical significance of the performance differences between CAMSDA-RMM and other methods, a paired *t*-test was conducted. The results for the second transfer scenario are presented in Table 4.

**Table 4:** Paired *t*-test results for the second transfer scenario.

Method	*t-*statistic	*p-*value
Single Best	5.7751	0.0022
JAN	3.3784	0.0197
DCTN	4.4817	0.0065
M3SDA	2.5875	0.0490
CASR	2.5005	0.0545
TSCDA	0.9575	0.3823

The results indicate that CAMSDA-RMM outperforms most baseline methods, with statistically significant improvements over Single Best, JAN, DCTN, and M3SDA (p < 0.05). The performance difference between CAMSDA-RMM and CASR approaches statistical significance (p = 0.0545), while the difference with TSCDA is not statistically significant (p = 0.3823). A combined analysis of both transfer scenarios suggests that CAMSDA-RMM is statistically superior to Single Best, JAN, DCTN, and M3SDA in both cases. Additionally, CAMSDA-RMM exhibits a significant advantage over CASR in the second scenario and over TSCDA in the first scenario.

### 4.4 Visualization analysis

**Fig 4:**
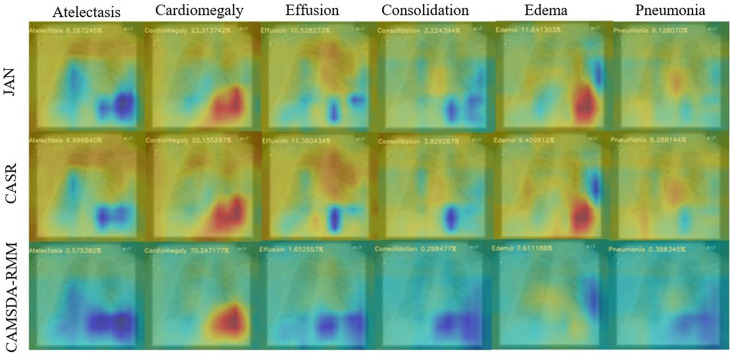
Comparison of the visualized results for three methods on Cardiomegaly disease in the first transfer scenario.

**Fig 5:**
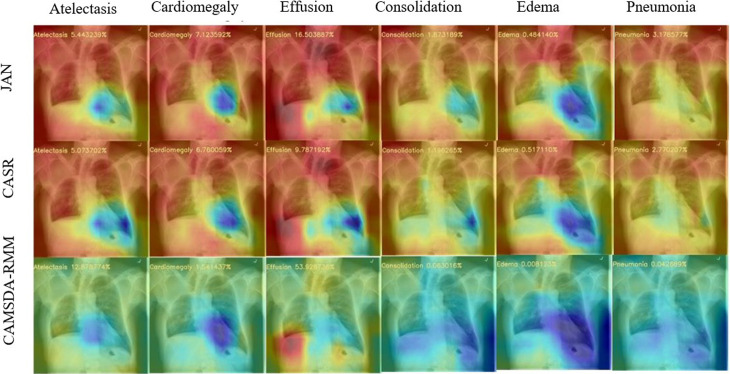
Comparison of the visualized results for three methods on Effusion disease in the second transfer scenario.

This section presents a visualization analysis for the two migration scenarios. The visualized samples selected in Fig 4 represent cases of Cardiomegaly, while those in Fig 5 correspond to Effusion. The comparative methods include JAN, CASR, and the proposed method. Class Activation Mapping (CAM) [28] is used to perform a visual ablation analysis on the chest X-ray images from the Open-i dataset. The background is blue, while the red or yellow areas indicate disease locations. The number in the top-left corner of each image represents the predicted probability for the corresponding disease.

As shown in Figs 4 and 5, the proposed method produces better visualization results compared to JAN and CASR. In Fig 4, both JAN and CASR fail to detect Cardiomegaly disease, while in Fig 4, both methods fail to detect Effusion disease. From the visualization results, it can be seen that JAN and CASR exhibit widespread yellow regions, which overlap with red areas, thus affecting their ability to accurately identify disease locations. In the case of Cardiomegaly in Fig 4 and Effusion in Fig 5, while JAN and CASR manage to mark the disease location with red, significant yellow areas in other regions distort their focus on the correct disease location. In contrast, the proposed method has more blue background regions, which clearly distinguish the background from the disease location, allowing for more accurate disease detection.

Furthermore, for diseases that are clearly absent, such as Consolidation, Edema, and Pneumonia in Figs 4 and 5, the extensive yellow regions in JAN and CASR result in more conservative predictions. However, the predicted probabilities of the proposed method are closer to 0 in these cases. The visualization results clearly demonstrate that the proposed method is more accurate.

### 4.5 Effectiveness analysis of the class-aware strategy

To validate the effectiveness of the proposed class-aware strategy in domain adaptation under the class shift scenario in multi-source domain adaptation, this section presents a comparative analysis of the optimizer parameter settings for the class-aware module, consistent with the settings in [23]. Using the first migration scenario as an example, the first method does not use the class-aware strategy, while the second method incorporates it. Each experiment is repeated five times to ensure result reliability, and the average classification accuracy is computed. The specific results are presented in Table 5, with bolded numbers representing the optimal values.

**Table 5:** The effectiveness analysis of the class-aware strategy in the first transfer scenario.

Method	AT	CA	EF	CO	ED	PN	Avg
Without strategy	90.00	96.89	96.72	87.55	94.38	89.75	92.55
With strategy	90.50	98.02	97.82	88.80	95.40	90.44	93.50

The experimental results show that without the class-aware strategy, the average classification accuracy is 92.55%. The specific accuracies for each disease are as follows: Atelectasis (90.00%), Cardiomegaly (96.89%), Effusion (96.72%), Consolidation (87.55%), Edema (94.38%), and Pneumonia (89.45%). When the class-aware strategy is applied, the average classification accuracy increases to 93.50%. The specific accuracies for each disease are as follows: Atelectasis (90.50%), Cardiomegaly (98.02%), Effusion (97.82%), Consolidation (88.80%), Edema (95.40%), and Pneumonia (90.44%). As shown in Table 3, after introducing the class-aware strategy, the model’s recognition accuracy for all diseases improves significantly. This enhancement is due to the class-aware strategy’s ability to effectively assist the model in capturing and adapting to the target domain’s class distribution features, thereby further optimizing classification accuracy. Therefore, the introduction of the class-aware strategy plays a crucial role in enhancing the overall performance of the model.

**Table 3:** Comparative analysis of CAMSDA-RMM with existing domain adaptation methods in the second transfer scenario.

Type	Method	AT	CA	EF	CO	ED	PN	Avg
Single source	Single Best	81.15	88.86	90.12	90.32	84.33	86.79	86.93
Multi-source	JAN	82.38	**91.97**	93.89	94.30	89.81	89.44	90.30
	DCTN	81.48	91.22	94.19	95.10	88.96	89.58	90.09
	M3SDA	83.09	87.20	**96.11**	95.10	86.87	87.40	89.29
	CASR	82.26	87.64	94.71	96.61	90.22	85.12	89.43
	TSCDA	82.98	92.13	95.88	95.76	**92.09**	88.68	91.25
	CAMSDA-RMM	**83.64**	91.50	95.30	**96.84**	91.88	**91.25**	**91.74**

### 4.6 Effectiveness analysis of the reweighted moment matching strategy

To validate the effectiveness of the proposed reweighted moment matching strategy for multi-source domain adaptation in the presence of class shift, this section compares the results of experiments using the same optimizer parameters as those in [21]. For the second transfer scenario, each experiment is repeated five times to ensure the reliability of the results, and the average classification accuracy is calculated, as shown in Table 6.

**Table 6:** Effectiveness of reweighted moment matching strategy in the second transfer scenario.

Method	AT	CA	EF	CO	ED	PN	Avg
Without strategy	82.88	90.90	95.70	96.51	91.36	90.22	91.26
With strategy	83.64	91.50	95.30	96.84	91.88	91.25	91.74

Experimental Results indicate that without the reweighted matrix matching strategy, the average classification accuracy is 91.26%. After incorporating the reweighted matrix matching strategy, the average classification accuracy improved to 91.74%. The recognition accuracy for most diseases increased, especially for Atelectasis and Pneumonia, which saw improvements of 0.76% and 1.03%, respectively. Although the accuracy for Effusion slightly decreased, the overall trend is clearly an improvement. The analysis reveals that, based on the class-aware strategy, which aligns the distributions of similar categories to enhance the forward transfer from source to target domains, the reweighted matrix matching strategy further optimizes the contribution of each source domain to the target domain. By adaptively updating the contribution weights, this strategy reduces errors in weight adjustment, leading to improved recognition accuracy. This experiment demonstrates that the reweighted matrix matching strategy has a significant effect in multi-source domain adaptation, effectively improving recognition accuracy in the target domain and validating its effectiveness in scenarios with class shift.

## 5. Conclusion

This paper addresses the issue of class shift in multi-source domain adaptation in medical scenarios by proposing an algorithm (CAMSDA-RMM) based on class awareness and reweighted matrix matching strategies. The algorithm introduces a class-aware strategy to effectively quantify the data distribution similarity between source and target domains, facilitating efficient knowledge transfer from source domains. Additionally, the reweighted matrix matching strategy dynamically constructs and adaptively updates the source domain contribution weights, precisely evaluating the roles of different source domains in the domain adaptation task. Experiments conducted on four publicly available chest X-ray datasets show that CAMSDA-RMM outperforms other advanced algorithms in solving complex multi-source domain adaptation problems, significantly improving model classification performance and generalization ability. It provides a reliable solution for multi-source domain adaptation in medical image analysis.

Future research will focus on developing more refined feature alignment methods to further enhance the performance of domain adaptation algorithms, while also tackling more complex class shift issues. This will promote the widespread application of multi-source domain adaptation algorithms in real-world medical scenarios.
